# Supplementary material for: Mutational meltdown of putative microbial altruists in Streptomyces coelicolor colonies
Source: Nat Commun. 2022 Apr 27;13:2266. doi: 10.1038/s41467-022-29924-y (PMC9046218; doi:10.1038/s41467-022-29924-y)
Supplement: Supplementary file 1 — Supplementary Information [file 41467_2022_29924_MOESM1_ESM.pdf]

Mutational meltdown of putative microbial altruists in *Streptomyces coelicolor* colonies

Zheren Zhang, Shraddha Shitut, Bart Claushuis, Dennis Claessen, Daniel E. Rozen

Supplementary Information

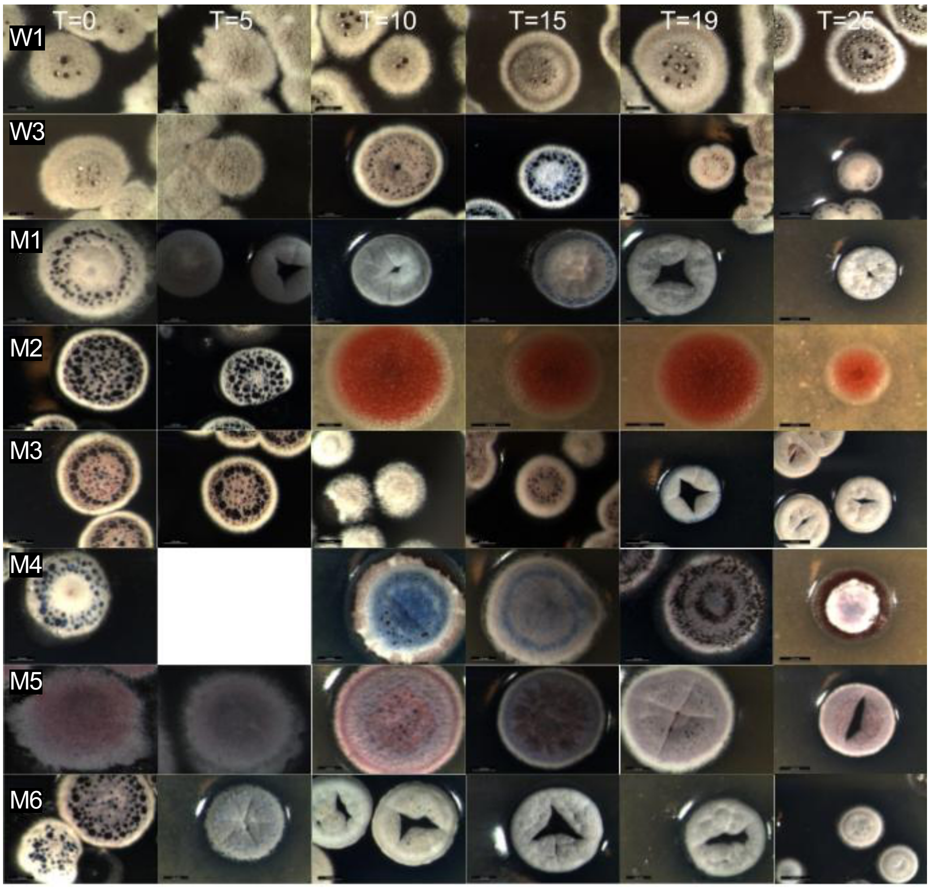

Supplementary Fig. 1 Morphology of sampled strains. Morphogenesis, including aerial growth, sporulation, and pigmentation, varies among and within lineages.

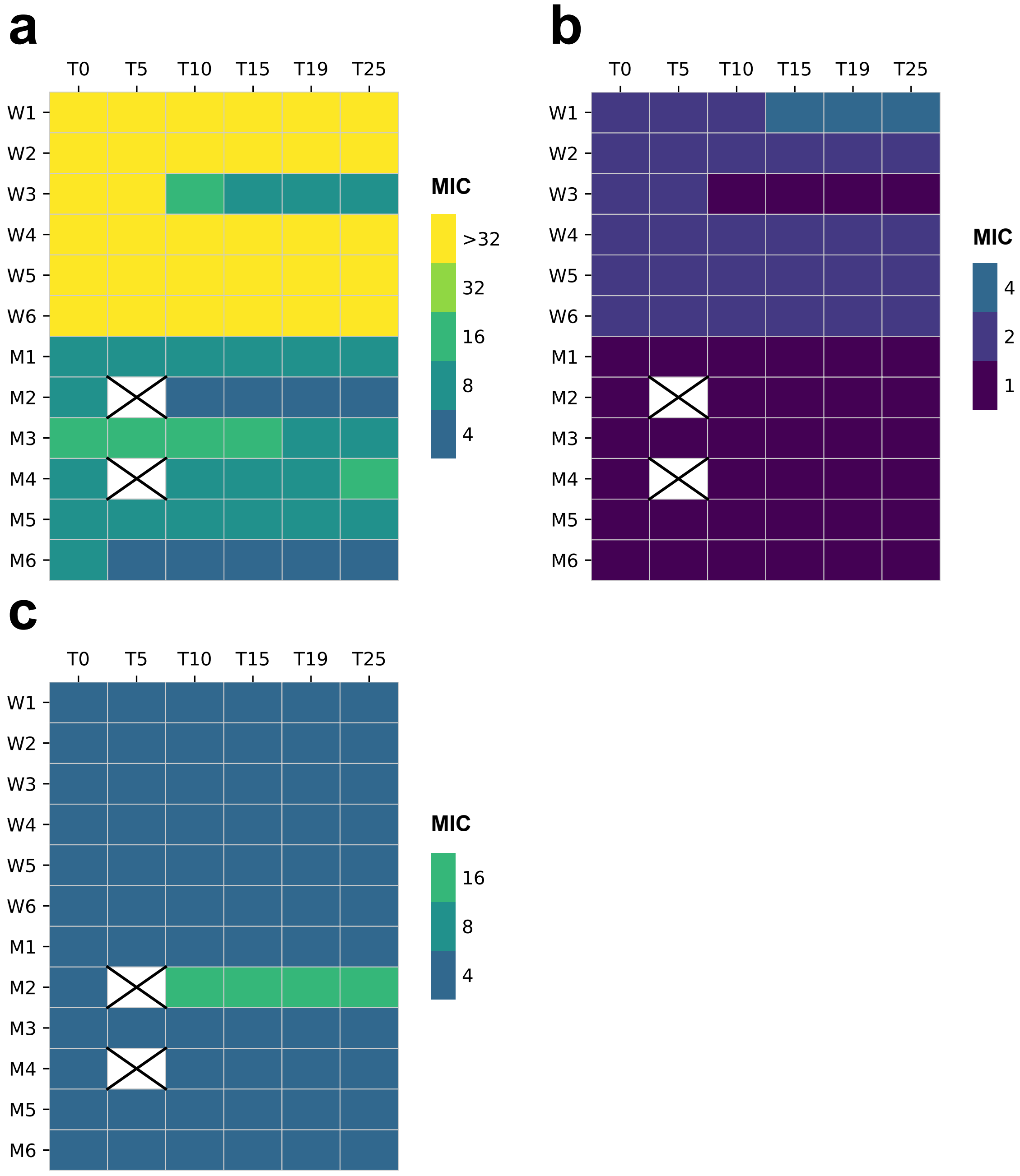

Supplementary Fig. 2 Antibiotic resistance of sampled strains. Graphs indicate the MIC of **a** oxytetracycline, **b** streptomycin, and **c** ciprofloxacin. The unit is shown as µg ml^{-1}.

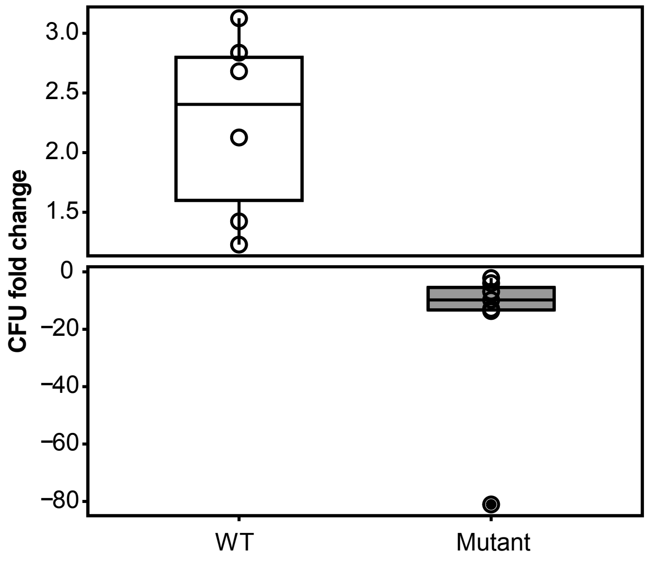

Supplementary Fig. 3 Changes of CFU production during the MA experiment. The CFU fold changes of WT (calculated from *n* = 6 lineages) and mutant (calculated from *n* = 7 lineages) lineages are shown. The box plot indicates the median (middle line), 25th, 75th percentile (box), minimum, maximum (whiskers), and outliers (black points) as well as individual data points (open circles).

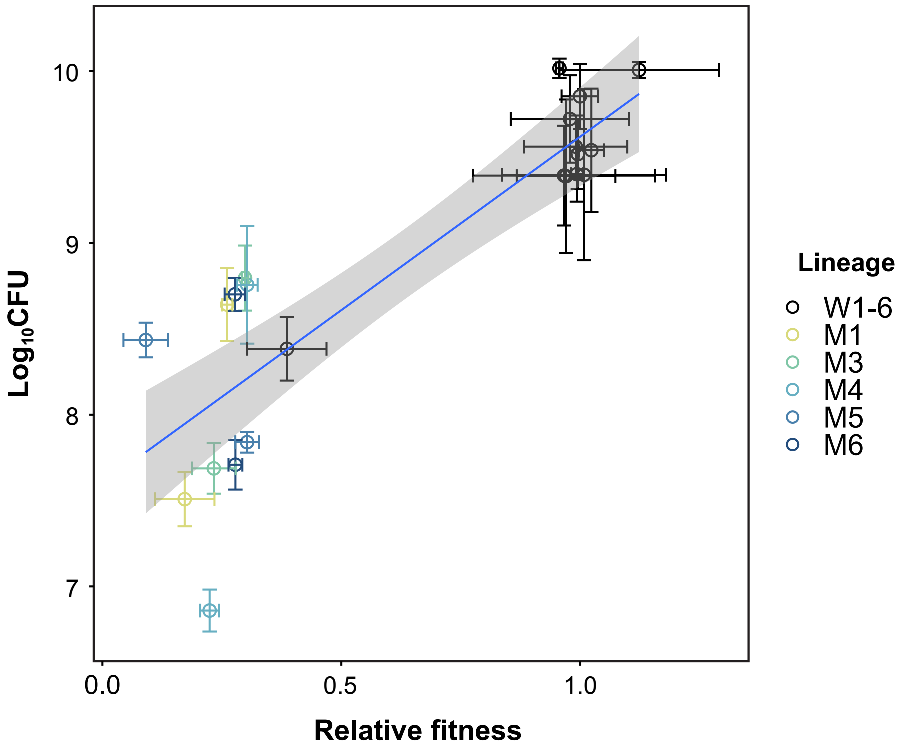

Supplementary Fig. 4 Correlation between relative fitness and CFU. A significant correlation is observed for sampled ancestral and evolved strains from different lineages. Data are from Fig. 3a, b and are represented as means with 95% confidence intervals. The linear regression line is included with the 95% confidence interval.

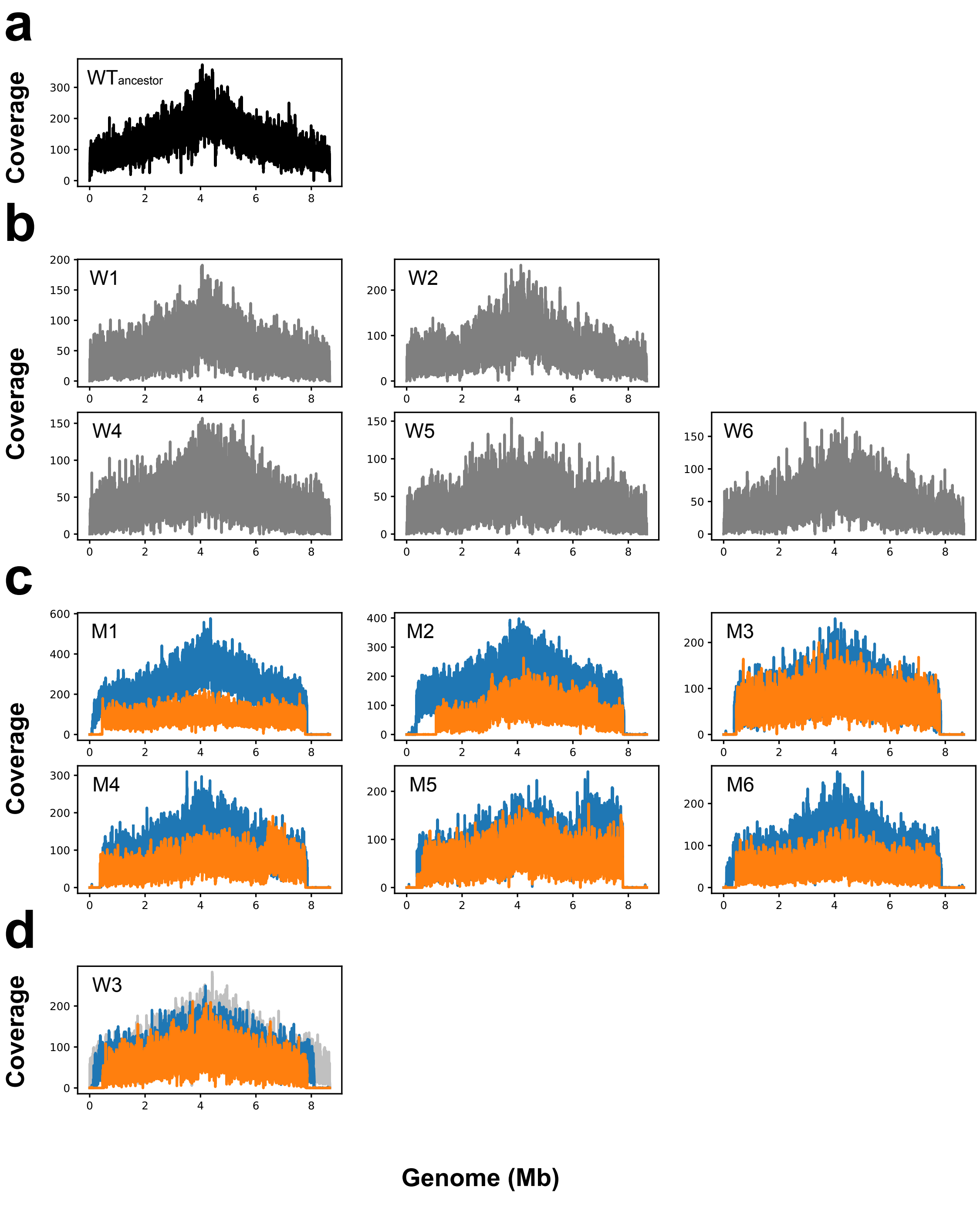

Supplementary Fig. 5 PacBio sequencing results of sampled strains. Graphs indicate the coverage of reads mapped to the *S. coelicolor* A3(2) M145 reference genome. Results of all sequenced strains are shown, including **a** the WT ancestor, **b** strains from WT lineages sampled at T0, **c** strains from mutant lineages sampled at T0 (blue) and T25 (orange), and **d** strains from lineage W3 sampled at T5 (light gray), T7 (blue) and T25 (orange).
