# Supplementary material for: Mutational meltdown of putative microbial altruists in Streptomyces coelicolor colonies
Source: Nat Commun. 2022 Apr 27;13:2266. doi: 10.1038/s41467-022-29924-y (PMC9046218; doi:10.1038/s41467-022-29924-y)
Supplement: Supplementary file 2 — Description of Additional Supplementary Information [file 41467_2022_29924_MOESM2_ESM.pdf]

Description of Additional Supplementary Files

Title: Supplementary Data 1

Description: Accumulated mutations in wild-type and mutant lineages during the experiment.
